# Determination of Deoxynivalenol Biomarkers in Italian Urine Samples

**DOI:** 10.3390/toxins11080441

**Published:** 2019-07-25

**Authors:** Barbara De Santis, Francesca Debegnach, Brunella Miano, Giorgio Moretti, Elisa Sonego, Antonio Chiaretti, Danilo Buonsenso, Carlo Brera

**Affiliations:** 1 Reparto di Sicurezza Chimica degli Alimenti, Istituto Superiore di Sanità, 00161 Rome, Italy; 2 Laboratorio di Chimica Sperimentale, Istituto Zooprofilattico Sperimentale delle Venezie, 35020 Legnaro (PD), Italy; 3 Istituto Zooprofilattico Sperimentale dell’Umbria e delle Marche, 06126 Perugia, Italy; 4 Dipartimento Scienze della Salute della Donna, del Bambino e di Sanità Pubblica, Fondazione Policlinico Universitario Agostino Gemelli IRCCS, Roma-Italia-Università Cattolica del Sacro Cuore, 00168 Roma, Italia

**Keywords:** mycotoxins, deoxynivalenol, children, adolescents, pregnant women, vegetarians, biomonitoring

## Abstract

Deoxynivalenol (DON) is a mycotoxin mainly produced by *Fusarium graminearum* that can contaminate cereals and cereal-based foodstuff. Urinary DON levels can be used as biomarker for exposure assessment purposes. This study assessed urinary DON concentrations in Italian volunteers recruited by age group, namely children, adolescents, adults, and the elderly. In addition, vulnerable groups, namely vegetarians and pregnant women, were included in the study. To determine the urinary DON, its glucuronide and de-epoxydated (DOM-1) forms, an indirect analytical approach was used, measuring free DON and total DON (as sum of free and glucuronides forms), before and after enzymatic treatment, respectively. Morning urine samples were collected on two consecutive days, from six different population groups, namely children, adolescent, adults, elderly, vegetarians and pregnant women. Total DON was measured in the 76% of the collected samples with the maximum incidences in children and adolescent age group. Urine samples from children and adolescent also showed the highest total DON levels, up to 17.0 ng/mg_creat_. Pregnant women had the lowest positive samples per category (40% for day 1 and 43% for day 2, respectively), low mean levels of total DON (down to 2.84 ng/mg_creat_) and median equal to 0 ng/mg_creat_. Estimation of DON dietary intake reveals that 7.5% of the total population exceeds the TDI of 1 μg/kg bw/day set for DON, with children showing 40% of individuals surpassing this value (male, day 2).

## 1. Introduction

Mycotoxins are natural food and feed contaminants, mainly produced by filamentous fungi of genera *Aspergillus*, *Penicillium*, *Fusarium* and *Alternaria* [1]. Mycotoxin production is promoted by either environmental and agronomic factors, while strong weather variability is a key factor for fungal infection, fungal colonization, and mycotoxin loads [2,3]. Among mycotoxins, trichothecenes represent the main group of *Fusarium* toxins commonly found in cereal grains. Deoxynivalenol (DON) is one of the most widely diffused natural-occurring trichothecene, is a sesquiterpenoid polar organic compound, belonging to the type B trichothecenes since it contains carbonyl group in C-8. *Fusarium graminearum* and *culmorum*, two important causing agents of the Fusarium Head Blight (FHB), are the most important producers of DON [4]. Acting as a virulence factor for cereal infection (namely in wheat, maize, barley, oat and rye), DON jeopardizes cereal grain quantity and quality from agriculture and health perspective [3] being persistent in cereal food derived products.

European Regulation (EC) 1881/2006 [5] has set maximum levels of DON in unprocessed cereals and cereal foods products intended for direct human consumption in the range of 1750 to 200 µg/kg. The scaled down levels of the contamination from the milling products and the processed ones reflect a well-known reduction rate of about 30% because of the cleaning and processing [6,7] but it has also to be considered the DON degradation process that occurs during industrial baking [8]. The legislative provisions serve to control the levels in food. However, surveys carried out worldwide [2] and in Italy [9] confirmed DON occurrence in cereal samples like wheat and maize, including processed products, thereby suggesting an exposure assessment issue. The correlation between DON dietary exposure and DON presence in urine has already been reported and confirmed [10,11]; therefore, the biomonitoring of DON and DON metabolites in urine may constitute a valuable indicator of the dietary exposure. On the basis of the available information derived from the toxicity studies, a temporary tolerable daily intake (TDI) of 1 μg/kg body weight (bw)/day was established in 2002 [12]. On the basis of the more recent available scientific data, this TDI has been recently confirmed by the European Food Safety Authority (EFSA) as a group-TDI for the inclusion of 3-AcDON, 15-AcDON and DON-3-glucoside, as DON plant metabolites [4]. In regards to acute risk, the Joint Expert Committee on Food and Additives (JECFA) [13] concluded that DON is a probable factor for acute pathologies in humans and derived an Acute Reference Dose (ARfD) of 8 μg/kg bw. In 2017, an extensive review on the presence of DON and its metabolites in human urine was carried out [14]. Authors showed that it is ubiquitous and highlighted geographical differences. So far, the metabolic profile of DON in animals has already been established. DON is metabolized to 13-deepoxy (DOM-1) in animals and is excreted via the feces and urine [15,16]. In addition to DOM-1, other conjugated products may be present as excreted metabolites, namely DON-3-glucuronide (DON-3GlcA) and DON-15-glucuronide (DON-15GlcA), which seem to be the major metabolites in humans, along with their iso forms and DON-8,15-hemiketal-8-GlcAc [17], sulfate and sulfonate forms [18,19]. More recently, DON-7-glucuronide (DON-7GlcA) has been tentatively identified and introduced as a new metabolite [20,21,22] in humans.

The analysis of urinary glucuronides is crucial for the study of trichothecene biomarkers, because approximately 80% of DON excreted via urine is conjugated with glucuronic acid [4]. A number of biomonitoring studies of DON in urine is available, however, different analytical method approaches, different limits of quantification and different ways to express measured mycotoxin (total DON or free DON, not specified, creatinine corrected, etc.) may give difficulties in comparison results. When expressed in ng/mg of creatinine (ng/mg_creat_), DON levels in urine ranged from 0.2 up to 903.7 ng/mg_creat_ [23,24,25,26,27,28], with a worst case reported for a group of pregnant women from Croatia [21].

In the present study, morning urine samples were collected over two consecutive days from 203 Italian volunteers; the participants were recruited by age group, namely children (3–9 years), adolescents (10–17 years), adults (18–64 years), and elderly (65 years or above). Vulnerable groups, namely vegetarians and pregnant women, were also included in the study. Each participant was asked to collect a first-morning urine sample on two consecutive days and to complete a Food Frequency Questionnaire (FFQ) reporting dietary habits over a 1-month period and a Food Diary (FD) with detailed information about food items consumed on the day preceding the collection of urine samples. Associations between food consumption and urinary DON were assessed using ordered logistic regression models.

To determine the urinary total DON expressed as sum of free and glucuronides forms, an indirect analytical approach, measuring free and total DON, before and after enzymatic treatment, respectively, was fully validated and applied to the collected urine samples [29,30].

This paper affords a distinctive data set detailing the DON exposure of Italian population in an urban setting, providing data on levels of DON in human urine samples collected, as analyzed by liquid chromatography coupled with mass spectrometry (LC-MS).

## 2. Results and Discussion

### 2.1. Analytical Method

The method used in this experimental study was previously published by Turner et al. [10] and applied with minor modifications. In particular, in order to reduce the amount of methanol needed for the elution of mycotoxins from the immunoaffinity column (IAC), three volumes were tested, 4 mL as recommended in the work of Turner et al., 2 mL and 1.5 mL. The performances were evaluated in terms of the recovery factor (*n* = 3), the obtained results were 102 ± 5%, 99 ± 5% and 56 ± 10%, for 4, 2 and 1.5 mL, respectively. The selected volume was 2 mL, for which a good recovery was achieved also permitting the reduction of the amount of organic solvent used and of the time needed for drying the sample in the subsequent analytical step. Since the IAC was designed for DON analysis, the IAC cross reactivity for DOM-1 was also tested (*n* = 3) with good results in terms of recovery (98 ± 1%). In regards to the determination step, a single injection for DON and DOM-1 was performed instead of the separated chromatographic run proposed in the reference paper [10].

### 2.2. In-House Validation

Before applying the method to the collected samples, an in-house validation was performed on 4 different contamination levels; the results obtained for the investigated performance parameters are reported in Table 1. The limits of detection and quantification (LoD and LoQ) values set during validation fit with the purpose of having an analytical method that is able to detect low amounts of DON and DOM-1 for exposure assessment purposes. Recovery factors are in the ranges 95–109% and 81–93% for DON and DOM-1, respectively, and the precision, evaluated in terms of Relative Standard Deviation of repeatability (RSDr), is always below 10%. Despite the absence of a specific official reference, the obtained values were considered satisfactory under the criteria of Regulation (EC) 401/2006 [31], which apply to any DON analysis on food. The average values estimated for method uncertainty ranged between 8 and 14% for DON and between 15 and 18% for DOM-1, being in both cases well below the 44%, which is the maximum acceptable value when the Horwitz approach is applied [31]. 

### 2.3. Description of Study Population

In total, 406 first morning urine samples were collected from 203 volunteers belonging to six population groups, namely children, adolescents, adults, elderly, vegetarians and pregnant women. All samples were kept at −20 °C until analysis. The volunteers enrolled for the study are reported, with their anthropometric data, grouped by category, gender and age in Table 2. 

The recruitment of subjects was subdivided between Istituto Superiore di Sanità (ISS) and the hospital “Agostino Gemelli (UCSC)”, with ISS responsible of the recruitment of adults, elderly and vegetarians and UCSC of children and adolescents by the clinics of Pediatric Unit and pregnant women from the clinics of Gynecology and Obstetrics outpatients. The selection of different groups of the population was specifically requested by EFSA while being considered as an asset for the study due to having different potential metabolic susceptibility, body weights, dietary habits, and consumption rates. Pediatric biological systems and detoxification processes might be widely different, causing an amplification of children’s susceptibility to hazards that would have negative consequences [32]. It should be noted that the cereal-based products consumption intakes figures of children in Italy are comparable with those of adults, showing, in addition, that children are consumers of a higher variety of those food products than adults (data from the FFQ and food diary, not shown). These two aspects provide children with, at least, the same exposure risk to that of adults. As for pregnant women, due to a decreased immunocompetence as a consequence of hormone levels changes and their complex and multifactorial interplay [33], this population group is of special interest for its possible susceptibility to DON, which has a recognized impact on the immune response system. As for vegetarians, they were included to attempt to verify and to measure if a special diet, which could be enriched by cereal based products, is a factor that may affect exposure estimates. However, the literature shows that these subgroups were already enrolled as special populations in other DON biomonitoring surveys. Piekkola et al., and Hepworth et al., [26,34] indicated the potential risk to mothers and their babies from DON exposure during pregnancy. Pestka highlighted the need to study the relationship between DON consumption and possible growth effects in susceptible populations such as children and vegetarians [35]. 

During the recruitment, UCSC initially encountered difficulties for pregnant women relating to a general concern from potential participants regarding the possible outputs of the study, abandonment of the study after initial recruitment, and incorrect collection of urine samples and compilation of questionnaires from participants. Therefore, ISS supported the UCSC hospital in its interactions with under-recruited subgroups (pregnant women and vegetarians). In regards to vegetarians, it was quite difficult to recruit male volunteers to the study since they constitute a low percentage of the vegetarian population.

The evaluation of the ethic aspects related to the study protocol and its approval by an Ethics Committee was requested and obtained before the starting date of the study. The UCSC ethical approval was granted by the Local Ethics Committee on 23 March 2014 (for the Gynecology Unit) and 7 April 2014 (for the Pediatric Unit). ISS ethical approval was granted by the ISS Ethic Committee on 18 February 2014. Informed consent was provided by the participants during their first visit. Approval code: Prot. PRE 84/14 and Prot. CE 14/413.

### 2.4. DON Biomarker Levels in Urine Samples

For each collected urine sample three analyses were performed, one for free and one for total DON and DOM-1, the first and second corresponding to before and after enzymatic treatment, with the third for creatinine. The results obtained are reported in Table 3, namely mean values, median (P50) and interquartile (IQR) values are listed as non-adjusted and creatinine-adjusted total DON. In Table 3 the percentage of samples above LOD for total DON is also reported for each category. Moreover, in Table 3 the percentage contribution of free DON and DON-GlcA to total DON is reported.

The mean DON level in urine for the total population studied was 7.67 and 7.93 ng/mL, but differences arose when the different categories were separately taken into account. Among the selected group, pregnant women had the lowest positive samples per category (40% for day 1 and 43% for day 2, respectively), low mean levels of total DON (4.37 ng/mL and 2.70 ng/mL on day 1 and day 2, respectively), and a median equal to 0 ng/mL. Conversely, urine from children and adolescents showed the highest concentrations of total DON, up to 75.9 ng/mL. The vegetarian group, apart from females on day 1, showed median values very close to the mean values, indicating that the values are close to normal distribution.

The median values of total DON in the morning urine ranged from 0 ng/mL for pregnant women on day 1 and day 2 to 12.60 ng/mL for adults male on day 2. However, when the creatinine adjustment was taken into account the median values ranged from 0 ng/mg_creat_ for pregnant women (day 1 and day 2) to 13.0 ng/mg_creat_ for children male (day 2), while the median value for adults male on day 2 decremented to 6.84 ng/mg_creat_. These results emphasize the importance of creatinine adjustment for the correct interpretation of results.

Regarding the DOM-1 content, only 6 samples out of the 406 analyzed urines (1.5%) had detectable levels of this toxin (ranges <LOD-2.6 ng DOM-1/mg_creat_ and <LOD-1.7 ng DOM-1/mg_creat_, for day 1 and day 2, respectively), confirming that this is a minor route for DON in human metabolism.

In view of the ambiguous statistical differences among males and females’ DON levels found in literature [11,30,36,37,38], DON ng/mg_creat_ content was checked between sexes in the general population excluding pregnant women. No statistical differences were highlighted for any of the age group (Mann-Whitney test). Conversely, a difference statistically significant was obtained comparing women adult group with pregnant women pointing out that pregnant women showed DON ng/mg_creat_ levels lower than adult females in general (*p*-value 0.025).

Comparing the results obtained within the EFSA study [39], the median concentrations of total DON adjusted for creatinine in morning urine in Italy and Norway were quite similar within similar population groups, whereas the median concentrations in the corresponding population groups in the UK were approximately 3-fold higher than in Italy and Norway. The Italian results are also in line with the values published by Solfrizzo et al. [30], which reported for total DON a median of 10.32 ng/mL.

Regarding the contribution of free DON and DON-GlcA to total DON, throughout all the population groups, the DON-GlcA represented 66% and 71% of the total DON for day 1 and day 2 respectively, confirming that the glucuronidation is an important route for DON excretion, as has already been reported in other studies [40].

### 2.5. Regression Analysis Between Food Consumption and DON Level in Urine Samples

The logistic regression model was applied to the dataset to assess the effect of the general variables on total DON concentration adjusted for creatinine. The output of the model refers to a unitary variation of the considered variable. With the aim to express the output of the food variable in a more comprehensive way, an increment of 10 grams of cereal food intake (i.e. total food, food category or item) was considered.

To assess the effect of the general variables, the mean values for the total DON content (ng/mg_creat_) on day 1 and day 2 were calculated for each subject and then categorized in tertiles, resulting in only one dependent variable. In regards to the age groups, the odds ratio (OR) to have a higher level of mean DON adjusted for creatinine in adults compared to children is 0.13 (*p* = 0.000), confirming the critical scenario for the children age group. The OR to have a higher level of mean concentration of total DON adjusted for creatinine in pregnant women compared to non-pregnant women is 0.198 (*p* = 0.001), in accordance with the very low DON levels in urine for this category. The gender, BMI, physical activity and vegetarian variables were not significant when added into the model.

Regarding the food variables, the analyses were performed by assessing the effect on total DON adjusted for creatinine on day 1 and day 2 of (i) total cereal food intake; (ii) each food group intake; (iii) single food items. While no statistically significant association between total food intake and DON levels was observed for day 1, the increase of 10 g in the total cereal food intake raised the OR by about 2.6% for day 2 (*p* = 0.027). The effects of the food groups provided significant results for pasta and pasta-like products, while for day 1 and day 2, an increased intake of 10 g caused the OR to have a higher level of concentration of total DON adjusted for creatinine by about 4% (*p* = 0.047) and 5.5% (*p* = 0.052), respectively. Considering the food item variables, the increased intake of durum wheat pasta caused the OR to have a higher level of total DON adjusted for creatinine by about 6% (*p* = 0.023) and 7.9% (*p* = 0.008), respectively.

### 2.6. Estimated DON Daily Intake

Starting from the total DON concentration levels measured in the collected urine samples, the Estimated Dietary Intake (EDI) was calculated using the following formula [30]

(1) $$EDI_{DON}=C\times \frac{V}{bw}\times \frac{100}{E}$$

EDI (ng/kg bw/day);
C = total DON concentration in the analyzed urine samples (ng/mL_urine_);
V = mean 24 h human urine volume (1.0 mL per kg of bw per hour for adults [41]; 2.0 mL per kg of bw per hour for children [42]);
bw = body weight reported in the questionnaire;
E = urinary excretion rate of DON in 24 h, 72.3% [10]

The calculated individual exposure followed a non-normal distribution (Shapiro-Wilk test) hence a non-parametric approach has been used. In Table 4, the mean, median (P50), and 95th percentile (P95) are reported. The obtained EDI values were compared with the Tolerable Daily Intake (TDI) set by EFSA for DON at 1000 ng/kg bw [4], in the last column of Table 4, the number of individuals and the percentage exceeding the TDI are reported.

The EDI mean values for the total population of the study are quite low, representing around the 30% of the TDI reported by EFSA, and the percentage went down to 20% when the median was considered. The scenario was more differentiated when the single categories were considered. The highest EDI values were obtained for children and to a minor extent for the adolescent age group, with mean values ranging from 577 to 937, and from 296 to 427 ng/kg bw/day for children and adolescent, respectively. Also, the number of individuals exceeding the TDI was higher for this age group, with the highest percentage being the 40% of the male children category on day 2. It is important to note that the two highest estimated EDI’s were obtained from female children on day 1 (5036 ng/kg bw/day) and male children on day 2 (3193 ng/kg bw/day), both values were below the ARfD (8000 ng/kg bw/day). As far as chronic exposure is concerned, all the values over the TDI should be duly considered as possible concern for public health as reported by EFSA, especially for vulnerable groups such as infants, toddlers and other children [4].

**Figure 1:**
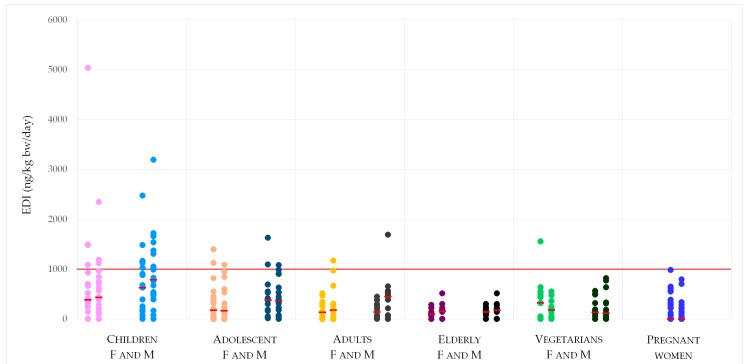
Distribution of the calculated individual EDI around the median value (red small line) and compared to the DON TDI (red line).

For a better visualization of the distribution of the obtained results, the calculated individual EDIs are reported in Figure 1. The depicted scenario for children and adolescent must be considered while taking into consideration body weights, homeostasis water balance (i.e., average food and beverage water input, urine feces, skin water output) and food consumption rates, especially for the children, that are not dissimilar when compared to the ones of the rest of the population, producing an unfavorable body weight/intake ratio.

The concentration values obtained in this study were also used by EFSA for exposure estimations. When comparing EFSA exposure estimates from biomarkers for adolescents, adult, elderly with corresponding mean averaged values obtained in this study (data not shown), exposure scenarios are confirmed. Discrepancies arise for children, and in particular, EFSA [4] obtained lower exposure estimates from biomarkers. These differences are due to different urine volumes considered for the 24 h, since EFSA used 0.5 L of urine for children during the 24 h, while in this study, calculations were made depending on the body weight of the subject [42], leading to a urine volume in the 24 h that ranged from 0.71 L/day to 2.49 L/day (an average of 1.25 L/day). The estimation made for the total Italian population in this paper, 329 and 343 ng/kg bw/day for day 1 and day 2 respectively, is also comparable with the probable daily intake (PDI) of 590 ng/kg bw/day reported by Solfrizzo [30], which was calculated considering a 50% excretion rate and 1.5 L for total urine in the 24 h for all of the recruited population.

## 3. Conclusions

The high incidence of DON in urine confirms its ubiquitous presence in cereal food products in Italy. However, the results of this study showed moderate mean levels of DON in urine samples of the studied cohort, despite differences that emerged when the different categories were considered, with children being the most susceptible group. The critical scenario depicted for children was also confirmed when the estimated daily intake was considered, showing the highest mean values for this age group (close to TDI and over in the case of P95), and the highest percentage of individuals exceeding the TDI, while for the total population the mean EDI represented around the 30% of the TDI. The higher exposure estimated for children and, to a minor extent for adolescents, can be explained by taking their unfavorable body weight/intake ratios into consideration.

In this study, the contribution of free DON and DON-GlcA to total urinary DON was also investigated and the results confirmed that DON-GlcA is the major DON metabolites for humans, on the other hand the very limited number of samples with DOM-1 above LOD confirms that this is a minor route for DON in the human metabolism.

Statistical analysis confirmed that age significantly affected urinary DON concentration (the difference between children and adults). Considering the outputs of the statistical model for food variables, increasing total cereal consumption was significantly associated with total DON in urine and in particular the increase of pasta consumption affected the urinary DON content more than the increased intake of other studied food items, confirming the relevant role of pasta in the Italian diet.

The results obtained in this study underpin the need for other studies in order to collect data for providing a more comprehensive exposure assessment of the Italian population. Moreover, support for the biomarker approach in exposure assessment is represented by the availability of either validated analytical methods and harmonized references for those critical figures (such as urine outputs or excretion rates), which sensibly influence the exposure scenarios. 

## 4. Materials and Methods 

### 4.1. Analytical Method

#### 4.1.1. Chemicals and Reagents

Methanol HPLC grade (Carlo Erba Reagenti, Cornaredo, MI, Italy) and ultrapure water (Millipore, Burlington, MA, USA) were used for the LC-MS analysis.

In order to perform the planned validation study and the analytical work on collected urine samples, the standard of DON (product number: D0156, 1mg), U-[^13^C_15_]-DON (99.0% ^13^C) and DOM-1 (product number: 34135, 2 mL) were purchased from Sigma (Saint Louis, MI, USA). The enzyme β-glucuronidase (Type IX-A from *E. coli*; product number: G7396—2MU) was purchased from Sigma too. The IAC DONtest WBTM (product number: G1066) were purchased from Vicam (Milford, MA, USA).

#### 4.1.2. Sample Preparation

Urinary DON and metabolites concentrations were measured using a two-step process. Stored urine samples were allowed to thaw, and were then centrifuged (1790 g, +4 °C, 15 min). For each participant, two aliquots (1 mL) were prepared by mixing ^13^C-DON internal standard solution, to provide a final concentration of 20 ng/mL.

Aliquot 1 was used to determine total DON concentrations, defined as the sum of glucuronide metabolites and free DON. To measure DON-glucuronides and free DON, each sample was adjusted to pH 6.8 with drop wise addition of KOH or HCl and digested using β-glucuronidase solution (23,000 U/mL, 250 μL) in a shaking water bath at 37 °C for 18 h, ensuring a gentle mixing. After this pre-treatment, the samples were diluted to a final 4 mL with phosphate buffered saline (PBS, pH 7.4). The diluted urine sample was passed through a wide bore DON IAC using a Visiprep^TM^ vacuum manifold (Sigma-Aldrich, Saint Louis, MI, USA). DON was eluted from columns with methanol (2 mL) and extracts were dried at 40 °C under a gentle stream of nitrogen and reconstituted in 90% methanol (250 µL) before LC-MS analysis. DOM-1 was quantified on the same aliquot and analyzed for DON-GlcA. Aliquot 2 was used to assess free DON using the aforementioned procedure, but without any β-glucuronidase treatment.

Urine creatinine was assessed by the enzymatic method described by Mazzachi et al. [43].

#### 4.1.3. LC-MS Determination

Chromatographic separation of DON and DOM-1 was performed using UHPLC (Ultra-High-Performance Liquid Chromatography) with Waters RP Acquity BEH C18 column (100 × 2.1 mm, 1.7 μm, Milford, MA, USA) kept at 30 °C, and a mobile phase sequence of 10 minutes duration, starting with 20% MeOH, changing to wash of 75% MeOH after 4.50 min and reverting to 20% MeOH after 8 min (flow rate 0.350 mL/min with a volume injection of 10 μL). DON elutes at 2.5 minutes under these conditions, while DOM-1 elutes at 4.5 min.

The mass spectrometric analysis was carried out with a Quattro Premier XE (Waters Milford, MA, USA) in SIR (Selective Ion Recording) acquisition mode with an ESI (ElectroSpray Ionization) interface. The analysis was performed in positive ion-mode. The following mass spectrometer conditions were optimized by direct infusion of DON and DOM-1 standard solutions: capillary voltage 4.0 kV, desolvation gas flow rate 500 L/h at 350 °C, source temperature 110 °C, cone voltage 40 V. The monitored masses for DON were 319.2 and 334.2 m/z corresponding to [DON-Na]^+^ and [^13^C-DON-Na]^+^ respectively, and 303.2 m/z for [DOM-1-Na]^+^.

For quantification purposes, calibration curves with a labeled internal standard for DON, and an external standard approach for DOM-1 were used. The curves covered the range 2–250 ng/mL, corresponding to 0.50–62.5 μg/L_urine_ for DON, and 2–200 ng/mL corresponding to 0.50–50 μg/L_urine_ for DOM-1. An acceptability criterion of *R*^2^ > 0.995 was applied during routine analysis.

DON-GlcA values were estimated indirectly by subtracting free DON values from total DON values for each analyzed urine sample.

### 4.2. In-House Validation

The in-house validation was performed in accordance with the Eurachem guideline [44]. Selectivity and specificity were guaranteed by the clean-up step with the IAC containing specific antibodies for the selected mycotoxins. LoD and LoQ were identified by the injection of diluted standard solutions, the requirements were a S/N = 3 for LOD and a S/N = 10 for LoQ. The LoQ was included in the validated contamination levels. The validation was performed on 4 different contamination levels for DON and DOM-1 by repeated analyses on spiked urine samples. Trueness was evaluated in terms of recovery factors, while precision was estimated in terms of Relative Standard Deviation of repeatability (RSD_r_) calculated on repeated analyses for each contamination level. Expanded uncertainty was also estimated by a metrological approach in accordance with Eurachem guideline [45]. The combined standard uncertainty was calculated by a metrological approach by summing up the standard uncertainty contributions from repeatability (Type A), recovery (Type A), pipetting volumes (Type B), and calibration (Type A). By applying a coverage factor of 2, the expanded uncertainty accounted for the 95% confidence interval. 

### 4.3. Study Design

The dataset in the present analysis represents a subset of data collected for a larger study entitled “Experimental study of deoxynivalenol biomarkers in urine” conducted for the European Food Safety Authority (EFSA) GP/EFSA/CONTAM/2013/04 [39]. In brief, this study explored the occurrence of DON and its metabolites in urine from different population groups (children, adolescents, adults, elderly, and pregnant women; total *n* = 635) in three European countries (UK, Italy, and Norway) and the relationships between urinary DON levels and its metabolites and self-reported dietary intake of cereal-based food items.

#### Recruitment of Participants and Urine Sample Collection

A target sample size of at least 200 individuals was established. The population groups included in the study were divided according to the age groups used within the EFSA Comprehensive European Food Consumption Database [46]. A relatively higher number of potentially more susceptible population groups such as children, adolescents, vegetarian and pregnant women was planned to be included. The planned sampled population included six different subgroups for recruitment, i.e., children (aged 3–9, 20%), adolescents (aged 10–17, 20%), adults (aged 18–64, 10%), elderly (aged above 65, 15%), vegetarians (15%) and pregnant women (20%).

Exclusion criteria included subjects not being able to give informed consent or complete the questionnaire, individuals affected by acute pathologies and any chronic illness (chronic renal, hepatic or cardiac problems, cancer), with chronic gastrointestinal conditions (e.g., celiac disease), gluten sensitivity or eating disorders, such as food allergies and those subjects recently on a weight loss diet, depression and psychosis, or hospitalized subjects within 3 months of admission. Inclusion criteria at the specialized recruitment centers (hospitals, clinics, institutions) required only healthy people, either not being on any medication or stable medication (for more than three months) that did not affect appetite (such as oral steroid use). As far as vegetarians, only people following the diet for >1 year and above the age of 18 years were recruited.

Collection equipment and instructions on how to collect and store a first morning urine sample were provided to participants. On two separate days, prior to urine sample collection, participants were required to complete a food diary, reporting food items consumed throughout those days. Participants provided a first morning urine sample the following morning. The urine samples (kept frozen at home) were returned to the clinical trial units after the second day of collection.

### 4.4. Regression Analysis Between Food Consumption and DON Levels in Urine Samples

#### 4.4.1. Food Frequency Questionnaire and Food Diary

Since a rich cereal-based diet plays a key role in the DON exposure, a semi-quantitative Food Frequency Questionnaire (FFQ) was designed. Type, frequency and quantity of the food consumed were obtained by this FFQ which has been prepared based on a validated questionnaire used in a Spanish study targeted to pregnant women [47]. The adopted FFQ included information about portion size and usual food frequency intake, with a recall period up to one month. In order to make the compilation of the questionnaire easier, photographic examples of portion sizes were also provided to participants. The food list in the adopted FFQ included specific food categories containing all food sources susceptible to DON contamination such as wheat, maize and barley products with emphasis on breads (whole meal, white, soft grain, other), breakfast cereals (high-fiber and other), pasta, pizza, fruit pies, biscuits, buns/cakes and beer. The reported food items reflected the specific Italian food habits. Therefore, in order to get information on the dietary habits of each population group, the FFQ was designed for gathering information on the amount and type of cereals and cereal-based products commonly more frequently consumed by the volunteers in the month prior the study and to capture and verify the most common food sources of DON in the diet. In order to capture such food categories, the EFSA’s Food Classification System FoodEx2 database was used [46].

Beside the FFQ, a Food Diary (FD) was also prepared with the aim of collecting information on the intake of the same food items as those included in the FFQ, but referred to the two days immediately before the urine sampling.

A database collecting all the data related to the enrolled subjects concerning individual information (age, gender, BMI, and other information) and food intake was prepared.

#### 4.4.2. Statistical Analysis

Statistical analyses were carried out using STATA/SE 12.0. For descriptive statistics measures (e.g. mean, median) a substitution method was applied. Values below LOD were substituted with 0. Shapiro-Wilk test was used for testing of normality distribution. Two sample Wilcoxon rank sum test (Mann-Whitney test) was used to compare within categories (e.g., Male/Female or Vegetarian Y/N).

To assess the effect of all the observed variables on the total DON content (expressed as ng/mg_creat_) an ordered logistic regression model was used. As variables, the model used a selection of the information gathered from the questionnaires including general variables such as age, gender and BMI, and food variables, namely the different food items reported on the FFQ and the FD. The model was run matching independent variables with the dependent variable. The variables that were shown to be significant were matched together to assess how much of the observed phenomenon is explained by the variables considered. When variables were significant, also interactions were considered. In order to apply the ordered logistic regression model, a categorization in tertiles (33th, 66th, 100th) of the dependent variable, namely the total DON content (ng/mg_creat_) on day 1 and day 2 was performed.

The odds ratio (OR) is used to quantify the extent of the association of the examined variable on the total DON (ng/mg_creat_) values.

A variable is considered significant when the null hypothesis of the relative coefficient of the variable is null with a significance level set to 0.95 (1-alpha). For this purpose, the *p* value was used. When the *p* value is higher than 0.05, the examined variable is considered to not be significant, conversely if the *p* value is lower than 0.05 the examined variable is considered to be significant.

## Figures and Tables

**Table 1:** Performance characteristics obtained during the in-house validation of the method.

Validation Parameter	DON	DOM-1
LoD; µg/L	0.25	0.25
Level 1 (LoQ); µg/L_urine_	0.50	0.51
Mean value; µg/L_urine_	0.54	0.43
s_r_; µg/L_urine_	0.03	0.03
RSD_r_; %	5.55	6.98
Recovery; %	109	84
Uncertainty; %	14	15
Level 2; µg/L_urine_	2.50	2.53
Mean value; µg/L_urine_	2.63	2.11
s_r_; µg/L_urine_	0.10	0.14
RSD_r_; %	3.80	6.63
Recovery; %	105	85
Uncertainty; %	8	18
Level 3; µg/L_urine_	12.50	12.63
Mean value; µg/L_urine_	12.16	11.65
s_r_; µg/L_urine_	0.33	0.37
RSD_r_; %	2.71	3.18
Recovery; %	95	93
Uncertainty; %	8	17
Level 4; µg/L_urine_	62.50	50.50
Mean value; µg/L_urine_	63.35	40.49
s_r_; µg/L_urine_	3.86	0.48
RSD_r_; %	6.09	1.19
Recovery; %	101	81
Uncertainty; %	9	14

**Table 2:** Anthropometric data and number of individuals recruited in Italy grouped by category, gender and age.

Group Category	Gender ^a^	N ^b^	Weight, kg	Height, cm	BMI ^c^, kg/m^2^
Children (3–9 years)	F	20	27	124	17.3
	M	20	26	119	17.2
Adolescent (10–17 years)	F	20	52	163	19.4
	M	20	63	173	20.7
Adults (18–64 years)	F	15	63	167	22.4
	M	16	77	179	24.0
Elderly (>65 years)	F	10	63	161	24.4
	M	9	70	170	24.3
Vegetarians	F	15	61	166	21.9
	M	16	75	176	24.9
Pregnant women	F	42	66	164	24.6

^a^ F, Female; M: Male; ^b^ N: number of subjects; ^c^ BMI, Body Mass Index.

**Table 3:** Non-adjusted and creatinine-adjusted total DON concentrations in urine samples by day and sex in sampled population groups, and contribution of free DON and DON-GlcA to the mean total DON concentration.

Age Group	Day	Gender ^a^	Samples Above LoQ (%)	Total DON (ng/mL_urine_)					Total DON (ng/mg_creat_)		
				Mean	P50	IQR	Free DON (%)	DON-GlcA (%)	Mean	P50	IQR
**Children (3–9 Years)**	1	F (20)	90	11.4	5.80	6.91	24	76	13.5	7.31	11.20
		M (20)	95	10.3	9.46	13.11	27	73	12.4	9.77	8.16
	2	F (20)	90	8.70	6.49	8.69	29	71	12.9	9.42	11.65
		M (20)	95	14.0	11.9	12.60	36	64	17.0	13.0	12.05
**Adolescents (10–17 Years)**	1	F (20)	80	9.83	5.36	11.89	24	76	9.38	6.20	11.37
		M (20)	100	12.9	11.4	12.71	34	66	15.6	9.30	17.54
	2	F (20)	85	9.19	5.12	7.40	15	85	12.6	8.06	9.74
		M (20)	90	12.3	11.0	12.17	30	70	11.3	9.96	11.89
**Adults (18–64 Years)**	1	F (15)	80	5.34	4.18	7.42	44	56	5.33	3.41	6.83
		M (16)	88	5.35	5.08	7.12	58	42	4.30	3.76	4.20
	2	F (15)	80	9.01	5.44	7.65	23	77	8.35	5.54	5.87
		M (16)	81	11.8	12.6	13.94	45	55	7.60	6.84	11.81
**Elderly (>65 Years)**	1	F (10)	70	5.07	3.18	6.01	41	59	6.69	5.13	6.10
		M (10)	90	4.93	4.40	4.10	41	59	6.37	6.87	8.79
	2	F (10)	60	5.51	4.40	3.25	35	65	7.79	5.16	3.84
		M (10)	80	6.16	5.59	3.40	20	80	12.4	8.09	3.84
**Vegetarians**	1	F (15)	80	10.6	9.74	14.74	19	81	16.1	10.1	30.75
		M (15)	73	4.10	3.30	5.22	35	65	4.43	3.23	7.85
	2	F (15)	80	5.53	4.17	5.34	23	77	8.47	8.25	12.73
		M (15)	73	5.88	3.03	7.79	30	70	4.28	2.25	7.44
**Pregnant Women**	1	F (42)	40	4.37	0.00	8.20	28	72	6.30	0.00	6.87
	2	F (42)	43	2.70	0.00	3.10	33	67	2.84	0.00	4.91
**Total**	1	(203)	76	7.67	4.58	8.63	34	66	9.21	5.05	9.81
	2	(203)	75	7.93	5.29	9.26	29	71	9.03	6.07	10.41

^a^ F: Female; M: Male; in parenthesis, number of subjects.

**Table 4:** Mean, median (P50) and 95th percentile (P95) of EDI (ng/kg bw/day) calculated for DON are reported based on the category, gender and age group, together with the number and percentage of individuals exceeding the TDI.

Age Group	Day	Gender	Mean	P50	IQR	P95	>TDI
**Children (3–9 Years)**	1	F (20)	757	385	459	3265	4 (20%)
		M (20)	683	628	870	1975	6 (30%)
	2	F (20)	577	431	577	1765	3 (15%)
		M (20)	937	791	837	2457	8 (40%)
**Adolescents (10–17 Years)**	1	F (20)	328	178	393	1261	2 (10%)
		M (20)	427	379	421	1359	1 (5%)
	2	F (20)	296	170	246	1019	1 (5%)
		M (20)	409	367	403	1030	1 (5%)
**Adults (18–64 Years)**	1	F (15)	177	139	247	513	0
		M (16)	170	168	237	446	0
	2	F (15)	299	181	254	1172	1 (7%)
		M (16)	411	416	463	1685	1 (7%)
**Elderly (>65 Years)**	1	F (10)	104	124	132	284	0
		M (10)	137	141	136	299	0
	2	F (10)	169	146	212	515	0
		M (10)	181	189	129	515	0
**Vegetarians**	1	F (15)	360	323	472	1555	1 (7%)
		M (15)	186	112	194	561	0
	2	F (15)	181	138	174	549	0
		M (15)	235	119	259	815	0
**Pregnant Women**	1	F (42)	154	0	271	630	0
	2	F (42)	96	0	103	519	0
**Total**	1	(203)	329	170	286	1144	14 (7%)
	2	(203)	343	181	310	1128	15 (8%)
